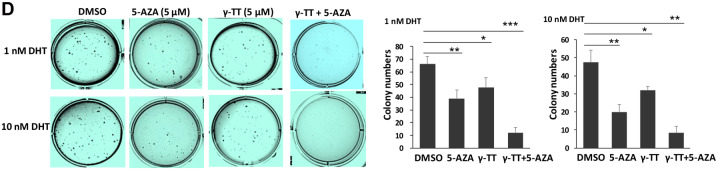# Corrigendum to ‘New therapy targeting differential androgen receptor signaling in prostate cancer stem/progenitor vs. non-stem/progenitor cells’

**DOI:** 10.1093/jmcb/mjaa077

**Published:** 2020-08-08

**Authors:** Soo Ok Lee, Zhifang Ma, Chiuan-Ren Yeh, Jie Luo, Tzu-Hua Lin, Kuo-Pao Lai, Shinichi Yamashita, Liang Liang, Jing Tian, Lei Li, Qi Jiang, Chiung-Kuei Huang, Yuanjie Niu, Shuyuan Yeh, Chawnshang Chang

**Affiliations:** 1 George Whipple Lab for Cancer Research, Department of Pathology, University of Rochester Medical Center, Rochester, NY 14642, USA; 2 George Whipple Lab for Cancer Research, Department of Urology, University of Rochester Medical Center, Rochester, NY 14642, USA; 3 George Whipple Lab for Cancer Research, Department of Radiation Oncology, University of Rochester Medical Center, Rochester, NY 14642, USA; 4 George Whipple Lab for Cancer Research, The Wilmot Cancer Center, University of Rochester Medical Center, Rochester, NY 14642, USA; 5 Department of Urology, First Hospital of Shanxi Medical University, Taiyuan 030001, China; 6 Sex Hormone Research Center, Department of Urology, The First Affiliated Hospital, Xi an Jiaotong University, Xi an 710061, China; 7 Chawnshang Chang Sex Hormone Research Center, Tianjin Institute of Urology, Tianjin Medical University, Tianjin 300211, China; 8 Sex Hormone Research Center, China Medical University and Hospital, Taichung 404

*Journal of Molecular Cell Biology* (2013), 5(1), 14–26, https://doi.org/10.1093/jmcb/mjs042

In this article, there were errors in the images presented in Figure 3Ab (bottom left) and Figure 5D (2nd and 4th columns). These were caused by miss-insertion of the images during preparation of the manuscript for submission. Since raw data of the images in Figure 5D were lost, the authors re-performed the whole experiment and provided new similar results (both images and quantitation graphs) for an update. The corrected Figure 3Ab left panel and updated Figure 5D are shown as below. The details have been corrected only in this corrigendum to preserve the published version of record. The authors assure that the original results or conclusions are not altered and they apologize for the mistake.

**Figure 3Ab left panel:**
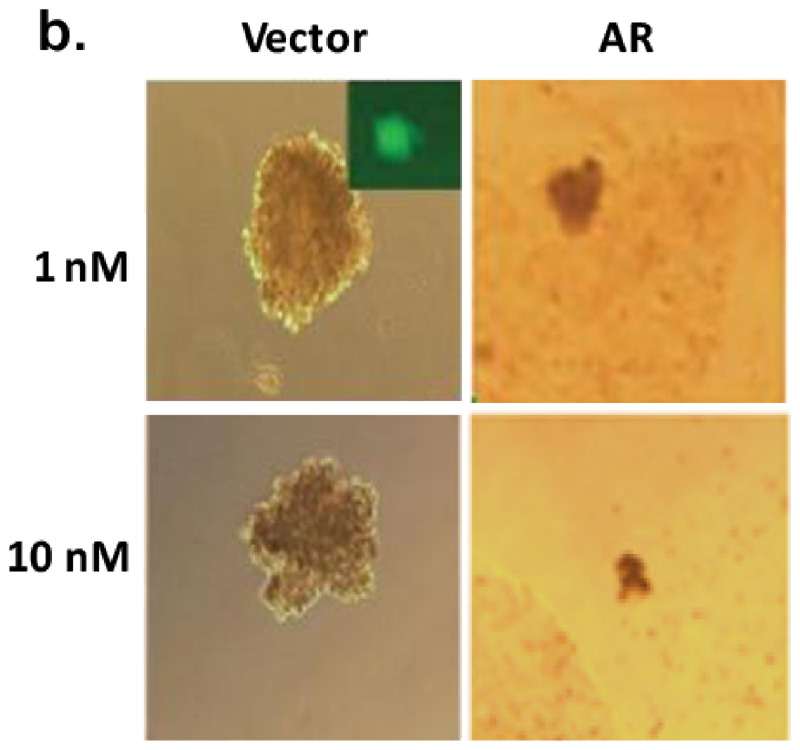


**Figure 5D:**